# The effect of heavy menstrual bleeding on sexual quality of life: a case-control study

**DOI:** 10.1590/1980-220X-REEUSP-2025-0041en

**Published:** 2026-09-25

**Authors:** Aynur Kizilirmak, Pelin Calpbinici, Nur Șifa Yavuz

**Affiliations:** 1 Nevsehir Hacı Bektaş Veli University, Semra and Vefa Küçük Faculty of Health Sciences, Department of Obstetrics and Gynecology Nursing, Nevsehir, Turkey.

**Keywords:** Sexuality, Menorrhagia, Women, Sexualidade, Menorragia, Mulheres

## Abstract

**Objective:**

To determine the effect of heavy menstrual bleeding (HMB) on the sexual quality of life of women.

**Method:**

A case-control study was conducted with a total of 142 women (71 with HMB and 71 controls) who applied to the outpatient clinics of a state hospital in Turkey. The case group consisted of women of reproductive age diagnosed with HMB, while the control group included women without HMB who met the inclusion criteria. Data were collected using a Personal Information Form and the Sexual Quality of Life-Female (SQOL-F).

**Results:**

There were no significant differences between the groups in terms of age and body mass index (BMI) (p > 0.05). However, compared to the control group, women with HMB reported a greater number of bleeding days, menstrual clots, higher pad/tampon usage, greater bleeding volume, menstrual discomfort, and shorter menstrual cycles (p < 0.05). A statistically significant difference was observed between the groups regarding educational level (p < 0.005), while no significant difference was found in contraceptive use (p > 0.05). The mean SQOL-F score was 71.91 ± 20.60 for women with HMB and 80.06 ± 17.39 for the control group, indicating a statistically significant difference (p < 0.05). Additionally, 26.8% of women with HMB had poor sexual quality of life.

**Conclusion:**

Although differences in educational level may represent a potential confounding factor, our findings demonstrate that HMB significantly reduces sexual quality of life in women, thereby supporting our research hypothesis.

## RESUMO

**Objetivo:**

Determinar o efeito do sangramento menstrual abundante (SMA) na qualidade de vida sexual de mulheres.

**Método:**

Estudo de caso-controle com um total de 142 mulheres (71 com SMA e 71 controles) atendidas nos ambulatórios de um hospital público na Turquia. O grupo de casos consistiu em mulheres em idade reprodutiva com diagnóstico de SMA. O grupo controle incluiu mulheres sem SMI que atenderam aos critérios de inclusão. Os dados foram coletados utilizando um Formulário de Informações Pessoais e a Escala de Qualidade de Vida Sexual Feminina (SQOL-F).

**Resultados:**

Não houve diferenças significativas entre os grupos em relação à idade e ao índice de massa corporal (IMC) (p > 0,05). Em comparação com o grupo controle, as mulheres com SMA relataram maior número de dias de sangramento, presença de coágulos menstruais, maior uso de absorventes externos/internos, maior volume de sangramento, desconforto menstrual e ciclos menstruais mais curtos (p < 0,05). Apesar da diferença estatisticamente significativa observada entre os grupos quanto ao nível de escolaridade (p < 0,005), não foi encontrada diferença significativa no uso de contraceptivos (p > 0,05). A pontuação média na SQOL-F foi de 71,91 ± 20,60 para mulheres com SMA e 80,06 ± 17,39 para o grupo controle, indicando uma diferença estatisticamente significativa (p < 0,05). Além disso, 26,8% das mulheres com SMA apresentaram baixa qualidade de vida sexual.

**Conclusão:**

Embora as diferenças no nível de escolaridade possam representar um potencial fator de confusão, nossos achados demonstram que o SMA reduz significativamente a qualidade de vida sexual das mulheres, corroborando assim a hipótese de pesquisa.

## INTRODUCTION

The International Federation of Gynecology and Obstetrics (FIGO) defines heavy menstrual bleeding (HMB) as excessive menstrual blood loss that affects quality of life related to physical, emotional, social and spiritual health^(1,2)^. Despite the absence of a comprehensive population-based study to ascertain the prevalence of HMB, various studies have documented prevalence rates ranging from 3% to 40.14%^(3, 4, 5, 6, 7, 8)^. According to the FIGO, HMB is classified etiologically into structural causes PALM (polyp, adenomyosis, leiomyoma, malignancy) and non-structural causes COEIN (coagulopathy, ovulatory dysfunction, endometrial, iatrogenic and unclassifiable)^(9)^. Although physical health problems such as excessive blood loss due to HMB, iron deficiency anemia and fatigue have been reported^(7)^, it has also been demonstrated to negatively affect women’s quality of life, energy level and mood. Furthermore, HMB has been linked to reduced work productivity, social interactions, family life, and quality of sexual life^(10,11)^.

The sexual life of women occupies a significant position within their overall lives. It is estimated that approximately 40% of women of reproductive age experience sexual health problems^(12)^. A multitude of factors can influence women’s sexual function, including mental health, sexual relationships, partner sexual function, personality-related factors, duration of the relationship, infertility, medications, chronic diseases, pelvic surgery, cancers, and postpartum changes^(13,14)^. An individual’s sexuality is significantly influenced by a multitude of factors, including their relationship, cultural and social environment, living conditions, biological characteristics, and self-perception. Additionally, hormonal fluctuations, menstruation, lactation, menopause, and multiple births can markedly influence women’s sexual functioning^(15,16)^. A review of the literature reveals that the terms “sexual quality of life”, “sexual satisfaction”, “sexual happiness”, and “sexual well-being” are used to convey the same underlying concept^(17)^. In this study, the term “quality of sexual life” will be employed in order to avoid any potential contradictions. The term “sexual quality of life” is defined as “an individual’s subjective evaluation of the positive and negative aspects of their sexual relationship and their subsequent emotional response to this evaluation”^(18)^. The sexual quality of life is associated with the physical, emotional, and relational aspects of sexuality. In light of these considerations, sexual pleasure, orgasm, and the frequency of sexual intercourse are regarded as being associated with the physical aspects of sexual quality of life^(17)^.

Heavy menstrual bleeding is known to be largely underreported. Studies indicate that only approximately 6% of women experiencing the condition seek medical care^(4,19)^, which may be attributed to misperceptions regarding HMB, the normalization of symptoms, and various cultural factors. In some cultures, menstrual bleeding is perceived as ‘medically cleansing’ or considered a sign of good health, while traditional norms of silence may hinder women from obtaining information about menstruation and seeking medical care for menstrual disorders by rendering such topics taboo^(20)^.

In most major religions worldwide, the belief that sexual intercourse should be avoided during menstruation is widespread^(21)^. The literature also supports this perspective, reporting that engaging in sexual intercourse during this period may lead to gynecological health issues such as endometriosis^(22)^ and infections^(23)^. Therefore, sexual activity is generally avoided during menstruation. In cases of HMB, the increased number of bleeding days and the heavier menstrual flow support the hypothesis that it may lead to a reduction in the frequency of sexual intercourse. Furthermore, the multicultural structure of Turkey and the presence of similar perspectives within Turkish culture further emphasize the significance of this study.

In this context, nurses play a critical role as the first point of contact with patients in the identification and management of HMB, distinguishing between normal and abnormal menstrual bleeding, providing education on menstrual care, and reducing menstruation-related taboos^(24,25)^. These contributions of nurses enhance women’s knowledge, facilitate access to appropriate care, and hold the potential to improve sexual quality of life.

Heavy menstrual bleeding does not merely impose a physical burden; it can also adversely affect health-related quality of life, social interactions, work productivity, and sexual life^(10)^. The existing literature includes studies addressing the impact of HMB and its related conditions on sexuality. In particular, the effect of uterine leiomyoma—one of the structural causes categorized under the PALM-COEIN classification—on female sexual function has been investigated^(26,27)^. Other studies have evaluated changes in female sexual function following medical and surgical treatments administered for abnormal uterine bleeding^(28, 29, 30)^. However, in the vast majority of these studies, female sexual function was assessed using the Female Sexual Function Index (FSFI). Although sexual function and sexual quality of life are closely interrelated, they constitute distinct concepts. While sexual function focuses on the physiological components of sexual response—such as desire, arousal, lubrication, orgasm, satisfaction, and pain^(31)^—sexual quality of life reflects an individual’s subjective satisfaction and psychosocial well-being regarding their sexuality^(20)^. A comprehensive search across the PubMed, Scopus, and Web of Science databases was conducted to identify the existing evidence in the literature. Combinations of the keywords ‘heavy menstrual bleeding’, ‘abnormal uterine bleeding’, ‘sexual quality of life’, ‘SQOL-F’, and related terms were used. Although several studies investigating HMB have evaluated female sexual function using the FSFI, no study assessing the impact of HMB on sexual quality of life using the Sexual Quality of Life–Female (SQOL-F) questionnaire could be retrieved. Furthermore, the promotion of women’s health is among the core priorities of the United Nations Sustainable Development Goals (SDGs). In particular, SDG 3 (Good Health and Well-being) and SDG 5 (Gender Equality) emphasize universal access to sexual and reproductive healthcare services (Targets 3.7 and 5.6). Determining the extent to which HMB affects women’s sexual quality of life can contribute both to the achievement of these global goals and to the development of more effective clinical interventions. Accordingly, this study aimed to determine the impact of HMB on women’s sexual quality of life.

The following hypotheses are presented for consideration:

H0: There is no difference in the quality of sexual life between women with and without HMB.
H1: There is a significant difference in the quality of sexual life between women with and without HMB.

## METHOD

### Study Design and Sample

A case-control study was conducted to ascertain the impact of HMB on the sexual quality of life of women. This study employed a prospective case-control design. The case group consisted of women of reproductive age, who applied to the Obstetrics and Gynecology Outpatient Clinic of a state hospital in a province of Turkey and were diagnosed with HMB by a qualified healthcare professional. To ensure privacy and confidentiality, data were collected in a quiet and private setting. Women in the HMB group completed the questionnaires in a private gynecological procedure room, whereas women in the control group completed them in a designated private room. Only the participant and the researcher were present during data collection, and the researcher was available solely to clarify any questions regarding the questionnaires. All questionnaires were completed before the participants’ consultation with the physician. Descriptive data were collected from participants using an information form based on ACOG criteria for HMB. The consistency of the findings with the ACOG definition indicates that participants in the case group were accurately identified. The control group consisted of women of reproductive age who applied to other outpatient clinics of the same hospital for various reasons, met the inclusion criteria, and had no diagnosis of HMB.

*Inclusion criteria:* sexually active women aged 18 years and older were included in the study. *Exclusion criteria:* menopausal women, those with mental disorders, with disabilities causing communication difficulties (such as hearing, speech, or language impairments) and without chronic diseases were excluded from the study. The sample size was calculated using the statistical analysis software OpenEpi Version 3.01 (http://www.openepi.com). A sample size of at least 64 women in each group was calculated using a 5% margin of error, 90% power, a 1:1 case-control ratio, a 3.5% odds ratio, and a 27.2% prevalence of HMB^(4)^. The requisite sample size was attained through the enrollment of 71 case and control women, allowing for the potential inclusion of missing individuals.

### Data Collection Tools

The researchers employed the Descriptive Information Form and the SQOL-F for data collection.

*Descriptive Information Form:* The researchers developed the form in accordance with existing literature on the subject. It consisted of 20 questions examining both sociodemographic characteristics (age, educational status, occupation, income status, etc.) and gynecological characteristics (age at first menarche, menstrual characteristics, contraceptive use, etc.).

*Sexual Quality of Life Scale-Female (SQOL-F):* The scale developed by Symonds et al.^(20)^ was adapted into Turkish by Tuğut and Gölbaşı^(32)^. This 6-point Likert-type scale has 18 items. Respondents are expected to answer each item based on their sexual experiences over the past four weeks. In the scoring system, each item is assigned a value between 1 and 6, allowing for a total score between 18 and 108 and a high score indicating a positive quality of sexual life. The measure is a useful instrument for assessing female quality of sexual life, with categorical ratings of poor (score range: 18–51), moderate (score range: 51–84), and good (score range: 84)^(33)^. In the Turkish adaptation of the scale, the Cronbach Alpha value was 0.83^(32)^. In the present study, the Cronbach’s Alpha value was determined to be 0.89.

### Data Collection

Data were collected between June 2023 and January 2024 through face-to-face interviews with participants in the case and control groups. Informed consent was obtained before data collection. The implementation period of the study lasted 10-15 minutes.

### Ethical Approval

Ethical approval was obtained from the Non-Interventional Clinical Research Ethics Committee of the relevant university before starting the research (Date: 27/03/2023, Decision no: 2023.05.09). Written authorization (Date: 24/05/2023, Decision no: E-26171210-929-216207373) was obtained from the pertinent institution to conduct the research. Furthermore, the women participating in the study were informed about the purpose, potential benefits, and the estimated time required for the interview. This information was provided before the interview and their written consent was obtained.

### Statistical Analysis

The data were analyzed using the IBM SPSS 25.0 (Statistical Package for the Social Sciences) software package. The statistical analyses included the calculation of percentage distributions, the determination of arithmetic means, the calculation of standard deviations, the implementation of t-tests in independent groups, the application of chi-square tests, and the completion of Cronbach’s alpha reliability analyses. The results were analyzed at a 95% confidence interval, and statistical significance was accepted at the p < 0.05 level.

## RESULTS

A total of 142 women were included in this study, comprising 71 cases of HMB and 71 controls. No statistically significant difference was observed between the mean age and body mass index (BMI) of the two groups (p > 0.05) (Table 1). The educational level of women in the control group was significantly higher, and a statistically significant difference in educational level was identified between the groups. (p < 0.05; Table 1).

**Table 1:** Comparison of socio-demographic characteristics between case and control groups (Nevşehir, Türkiye, 2024).

Variable	Case (n = 71)	Control (n = 71)	Statistical analysis	*p* value
	n (%)	n (%)		
**Age (Mean ± SD)**	44.41 ± 5.19	42.49 ± 7.26	1.809*	0.073
**Education level**				
Literate/primary school	38 (53.5)	21 (29.6)	15.024**	
Secondary school/high school	22 (31.0)	19 (26.8)		**0.001**
University	11 (15.5)	31 (43.7)		
**Income status**				
Income lower than expenses	5 (7.0)	16 (22.5)	9.008**	
Income equal to expenses	61 (85.9)	46 (64.8)		**0.011**
Income higher than expenses	5 (7.0)	9 (12.7)		
**BMI (Mean ± SD)**	75.28 ± 13.12	72.63 ± 13.45	1.188*	0.23

*t: independent t test

**X^2^: chi-square test

Women with HMB had significantly longer bleeding duration, more frequent menstrual clots, shorter menstrual cycle intervals, heavier menstrual bleeding, more frequent troublesome excessive bleeding during menstruation, more blood leakage on the bed or underwear, and more intermenstrual bleeding than women in the control group (all p < 0.05; Table 2).

**Table 2:** Comparison of menstrual signs and symptoms between women with and without HMB (Nevşehir, Türkiye, 2024).

Variable	Case (n = 71)	Control (n = 71)	Statistical analysis	*p* value
	n (%)	n (%)		
**Menarche age (Mean ± SD)**	13.29 ± 1.35	13.35 ± 1.08	–0.275	0.784
**Number of bleeding days**				
1–7	41 (57.7)	62 (87.3)	15.589	**<0.001**
8 or more	30 (42.3)	9 (12.7)		
**Menstrual blood clots**				
Yes	55 (77.5)	27 (38.0)	22.628	**<0.001**
No	16 (22.5)	44 (62.0)		
**Menstrual cycle length (days)**				
8–24	46 (64.8)	16 (22.5)	25.766	**<0.001**
24 and above	25 (35.2)	55 (77.5)		
**Menstrual bleeding amount**				
Heavy	48 (67.6)	7 (9.9)	28.873	**<0.001**
Moderate	23 (32.4)	61 (85.9)		
Light	–	3 (4.2)		
**Troublesome excessive bleeding during menstruation**				
Yes	54 (76.1)	19 (26.8)	34.534	**<0.001**
No	17 (23.9)	52 (73.2)		
**Blood leakage on bedding or underwear**				
Yes	55 (77.5)	21 (29.6)	32.726	**<0.001**
No	16 (22.5)	50 (70.4)		
**Intermenstrual bleeding**				
Yes	35 (49.3)	10 (14.1)	20.332	**<0.001**
No	36 (50.7)	61 (85.9)		

Women with HMB also reported using a greater number of sanitary pads or tampons per day, more frequent use of double sanitary protection, and more frequent waking at night to change sanitary protection than women in the control group (all p < 0.05; Table 3). No statistically significant differences were observed between the groups regarding contraceptive use or contraceptive methods (p > 0.05; Table 3).

**Table 3:** Comparison of menstrual hygiene behaviors and contraceptive characteristics between women with and without HMB (Nevşehir, Türkiye, 2024).

Variable	Case (n = 71)	Control (n = 71)	Statistical analysis	*p* value
	n (%)	n (%)		
**Number of pads or tampons/day**				
1–4	41 (57.7)	56 (78.9)	7.320	**0.006**
5–10	30 (42.3)	15 (21.1)		
**Use of double pads during menstruation**				
Yes	35 (49.3)	10 (14.1)	20.332	**<0.001**
No	36 (50.7)	61 (85.9)		
**Waking up at night to change pads during menstruation**				
Yes	48 (67.6)	20 (28.2)	22.124	**<0.001**
No	23 (32.4)	51 (71.8)		
**Contraceptive use**				
Yes	48 (67.6)	54 (76.1)	1.253	0.263
No	23 (32.4)	17 (23.9)		
**Contraceptive methods**				
Combined oral contraceptives / monthly injectable contraceptives/ Hormonal intrauterine device (LNG-IUD)	3 (6.3)	6 (11.1)	9.375	0.051
Copper IUD (non-hormonal)	13 (27.1)	11 (20.4)		
Male condom	18 (37.5)	12 (22.2)		
Tubal ligation	10 (20.8)	9 (16.7)		
Coitus Interruptus	4 (8.3)	16 (29.6)		

The mean SQOL-F score for women experiencing HMB was 71.91 ± 20.60, while the mean score for women without HMB was 80.06 ± 17.39. A significant difference was observed between the mean SQOL-F scores of the case and control groups (p < 0.05). A total of 19 (26.8%) women with HMB exhibited poor SQOL-F, 27 (38.0%) demonstrated moderate SQOL-F, and 25 (35.2%) exhibited good SQOL-F. A comparison of the case and control groups revealed that women with HMB exhibited a lower quality of sexual life (p < 0.05; Table 4).

**Table 4:** Comparison of the mean scores of the Sexual Quality of Life Scale in case and control groups (Nevşehir, Türkiye, 2024).

SQOL-F	Case (n = 71)	Control (n = 71)	Statistical analysis	*p* value
SQOL-F (Mean ± SD)	71.91 ± 20.60	80.06 ± 17.39	–2.548	**0.012**
Poor	19 (26.8)	8 (11.3)	6.465	**0.039**
Moderate	27 (38.0)	27 (38.0)		
Good	25 (35.2)	36 (50.7)		

## DISCUSSION

Although largely underreported^(4)^, in the literature, HMB is documented as being experienced by more than one-third of women of reproductive age^(7)^. Given the potential influence of age on both HMB and sexual quality of life, the age characteristics of the case and control groups in our study were similar. In the existing literature, studies on similar subjects consistently compare groups according to age characteristics^(34, 35, 36)^.

The higher proportion of university graduate women in the control group indicates a statistically significant difference in educational level between the groups, which may act as a potential confounding factor affecting women’s sexual quality of life. Education is a key determinant in shaping individuals’ sexual health, sexual desire, and levels of sexual activity^(37)^. Higher levels of education are associated with the development of healthier lifestyle habits, greater access to healthcare services, and increased likelihood of obtaining accurate information regarding sexual health^(38)^. Higher education is linked to enhanced communication skills, which may strengthen sexual communication and satisfaction within relationships^(39)^. However, higher educational attainment may also expose individuals to more stressful and demanding employment conditions, which can negatively affect sexual satisfaction due to increased stress and distracting thoughts^(40)^. Additionally, individuals with different educational levels may exhibit significantly different priorities and expectations regarding sexual activity^(41)^. A study of 300 married women evaluating the impact of education on female sexuality^(42)^ found a significant relationship between educational level and sexual quality of life. This finding aligns with a systematic review examining structural determinants of health, which similarly reported an association between education and women’s sexual quality of life^(43)^. Although clear boundaries between education and sexuality are difficult “to establish, individuals with higher education levels generally report better sexual health outcomes, possess a broader perspective on sexual well-being, and experience greater overall satisfaction with their sexual lives^(39)^. When the findings derived from the literature are considered collectively, it becomes evident that the effect of educational level on sexual quality of life is multidimensional and may manifest in varying forms depending on the individual’s living conditions, relationship dynamics, and cultural context. Consequently, the imbalance in educational distribution across groups in the present study emerges as a significant methodological consideration that warrants cautious interpretation of the findings obtained. Future research is recommended to address this issue through matching procedures or statistical control.

In 2021, the American College of Obstetricians and Gynecologists (ACOG) defined HMB as bleeding that persists for more than seven days, soaks through one or more tampons or pads every hour for several consecutive hours, requires wearing more than one pad at a time to control the flow, requires changing pads or tampons overnight, and includes blood clots that are a quarter in size or larger^(44)^. The case group consisted of women who had been diagnosed with HMB by a qualified medical practitioner. In our study, women with HMB exhibited a higher prevalence of the following symptoms compared to the control group: increased duration of bleeding, menstrual clots, use of pads/tampons per day, volume of menstrual bleeding, discomfort due to excessive bleeding during menstruation, use of double pads during menstruation, bleeding on the bed or underwear during menstruation, nocturnal pad changes during menstruation, and an increased frequency of menstruation. Our findings align with the ACOG^(44)^ definition of HMB. This suggests that the case group participants were correctly identified.

Our study revealed that women with HMB reported a higher prevalence of prolonged bleeding duration, increased bleeding volume, blood clots, use of double pads, nocturnal pad changes, and symptoms limiting social life compared to the control group. This finding is significant as the literature reports that HMB negatively affects women’s social, emotional, and psychological well-being, thereby directly and indirectly impairing sexual quality of life. Heavy menstrual bleeding restricts participation in social and leisure activities and limits physical activity due to the fear of leakage and the frequent need to change sanitary products^(45)^. These factors contribute to emotional distress, including low mood, anxiety, and reduced quality of life. Since emotional health is closely linked to sexual quality of life, the psychological burden associated with HMB may adversely affect sexual desire and satisfaction^(46,47)^. Moreover, the chronic nature of HMB may lead to long-term psychological stress, which can further negatively impact intimate relationships^(45)^. Accordingly, HMB should be regarded not merely as a physiological bleeding disorder, but as a comprehensive clinical entity that exerts measurable adverse effects on sexual health, emotional functioning, and relationship quality. In this regard, the clinical management of HMB necessitates the development and implementation of multidisciplinary intervention models that, alongside symptomatic treatment, explicitly target women’s sexual well-being and psychosocial health. Although women with HMB reported greater use of sanitary pads/tampons and more frequent nocturnal pad changes, these behaviors may also be influenced by individual hygiene practices, personal preferences, or the absorbency of sanitary products. Therefore, these variables should not be interpreted as independent indicators of menstrual blood loss, but rather as supportive behavioral findings considered alongside physician-confirmed HMB diagnosis and other clinical characteristics.

The subjective experience of HMB is shaped by cultural norms and societal expectations. In many cultures where menstruation is stigmatized, women may feel pressured to conceal their symptoms, which can negatively influence their participation in sexual activity and their willingness to discuss sexual health concerns with partners or healthcare professionals^(48)^. Menstrual taboos, feelings of shame, and perceptions of being ‘unclean’ can lead women to avoid sexual intimacy and hesitate to seek medical care, thereby negatively impacting both sexual quality of life and overall well-being^(48, 49, 50)^. This situation precipitates diagnostic delays and a prolonged symptom burden, contributing to the chronification of psychological distress and, consequently, to a further deterioration of sexual quality of life.

Heavy menstrual bleeding is closely associated with negative affect, social embarrassment, and diminished morale. The risk of heavy bleeding and the fear of leakage lead women to withdraw not only from social environments but also from intimate relationships, which may result in a weakening of sexual self-confidence and a reduction in the desire to engage in sexual activity^(49)^. Indeed, current literature demonstrates that HMB is experienced as a sexual constraint and exerts direct adverse effects on women’s sexual autonomy^(51)^. Furthermore, the social stigma specific to HMB renders it difficult for women to openly express this experience, constituting a barrier to sexual communication with both healthcare professionals and partners^(52)^. Consequently, HMB may substantially impair sexual quality of life not solely through physiological mechanisms, but also through profound social and emotional pathways.

The mean scores of the SQOL scale for both the case and control groups were found to be at an intermediate level. However, the mean score for the SQOL was observed to be lower in women with HMB compared to women without HMB. A review of the literature reveals a dearth of direct studies evaluating the quality of sexual life among women with HMB. Nevertheless, a meta-analysis of studies with women of reproductive age revealed that chronic disease status was a significant predictor of sexual quality of life^(13)^. Vallée et al.^(53)^ identified HMB as an independent and robust predictor of sexual dysfunction in women, reporting that its presence, in conjunction with severe menstrual cramping and the absence of treatment, significantly increases the risk of sexual dysfunction. In contrast, the systematic review conducted by Vitale et al.^(29)^ demonstrated that treatments targeting abnormal uterine bleeding yielded significant improvements in sexual quality of life and psychological well-being, thereby indirectly corroborating the adverse impact of untreated HMB on sexual quality of life. The study revealed that one in four women with HMB and one in 10 women without HMB had a poor level of sexual quality of life. Conversely, approximately one in three women with HMB and one in two women without HMB demonstrated a favorable level of sexual quality of life (Table 3). The findings of our research indicate that women experiencing HMB report a lower quality of sexual life than women who do not experience such bleeding. The findings of our study are in alignment with our initial research hypothesis.

Nurses play a critical role in the identification, management, and education related to HMB^(54)^. Assessing both the direct and indirect effects of HMB on sexual quality of life is an essential component of nursing care. By providing women with information about the causes, symptoms, and self-management strategies of HMB, nurses can help them distinguish between normal and abnormal menstrual cycles^(55)^. Supporting open communication about sexual health, offering referrals for psychological counseling, and providing guidance to reduce societal taboos are among the significant contributions of nurses to improving women’s sexual quality of life^(24,54,55)^. Therefore, nurses occupy a key position in mitigating the impact of HMB on sexual health.

### Strengths and Limitations of the Study

The strength of this study lies in the case group being diagnosed by a physician, supported by ACOG diagnostic criteria for HMB. However, limitations include matching groups solely by age and relying on self-reported questionnaire responses. Additionally, the statistically significant difference in educational level between groups may represent a confounding factor, influencing perceptions of sexual quality of life.

## CONCLUSION

Despite the statistically significant difference in educational level between the case and control groups and the consideration of this difference as a potential confounding factor affecting sexual quality of life, our findings demonstrate that women with HMB have a significantly lower sexual quality of life compared to those without HMB, thereby supporting our research hypothesis.

## Data Availability

The entire dataset supporting the results of this study is available upon request to the corresponding author

## References

[1] National Institute For Health and Care Excellence (2018). Heavy menstrual bleeding: assessment and management [Internet]. https://www.nice.org.uk/guidance/ng88.

[2] Sriprasert I, Pakrashi T, Kimble T, Archer DF (2017). Heavy menstrual bleeding diagnosis and medical management. Contracept Reprod Med.

[3] Abrahamsson K, Åkerman E, Ström S, Söderman L, Källberg H, Klingberg-Allvin M. (2025). Menstrual-related symptoms and absence from school among young people in Sweden: a stratified, randomized, population-based survey. BMC Public Health.

[4] Fraser IS, Mansour D, Breymann C, Hoffman C, Mezzacasa A, Petraglia F (2015). Prevalence of heavy menstrual bleeding and experiences of affected women in a European patient survey. Int J Gynaecol Obstet.

[5] Jain V, Munro MG, Critchley HOD (2023). Contemporary evaluation of women and girls with abnormal uterine bleeding: FIGO Systems 1 and 2. Int J Gynaecol Obstet.

[6] Kazemijaliseh H, Ramezani Tehrani F, Behboudi-Gandevani S, Khalili D, Hosseinpanah F, Azizi F (2017). A population-based study of the prevalence of abnormal uterine bleeding and its related factors among iranian reproductive-age women: an updated data. Arch Iran Med.

[7] Kocaoz S, Cirpan R, Degirmencioglu AZ (2019). The prevalence and impacts heavy menstrual bleeding on anemia, fatigue and quality of life in women of reproductive age: heavy menstrual bleeding,Anemia, Fatigue & QOL in women of reproductive age. Pak J Med Sci.

[8] Rafique N, Al-Sheikh MH (2018). Prevalence of menstrual problems and their association with psychological stress in young female students studying health sciences. Saudi Med J.

[9] Munro MG, Critchley HOD, Fraser IS (2018). The two FIGO systems for normal and abnormal uterine bleeding symptoms and classification of causes of abnormal uterine bleeding in the reproductive years: 2018 revisions. Int J Gynaecol Obstet.

[10] Singh SS, Calaf Alsina J, Vannuccini S, Koga K, Lopes Silva-Filho A, Yang X (2022). Clinical perspectives on the menstrual pictogram for the assessment of heavy menstrual bleeding. Hum Reprod Open.

[11] Lukes AS, Baker J, Eder S, Adomako TL (2012). Daily menstrual blood loss and quality of life in women with heavy menstrual bleeding. Womens Health.

[12] DeRogatis LR, Burnett AL (2008). The epidemiology of sexual dysfunctions. J Sex Med.

[13] Alidost F, Pakzad R, Dolatian M, Abdi F (2021). Sexual dysfunction among women of reproductive age: A systematic review and meta-analysis. Int J Reprod Biomed.

[14] Sabola NE, Shahin MA, Khader KA, Metwally HM, Desoky MM (2020). SExual nursing care for the postpartum period and its impact on sexual dysfunction and life satisfaction among Egyptian women. Malays J Public Health Med.

[15] American College of Obstetricians and Gynecologists (2019). Female sexual dysfunction: ACOG practice bulletin clinical management guidelines for obstetrician-gynecologists, number 213. Obstet Gynecol.

[16] O’Laughlin D (2026). Female sexual dysfunction: screening, diagnosis, and management. JAAPA.

[17] Dogan T, Tugut N, Golbasi Z (2013). The relationship between sexual quality of life, happiness, and satisfaction with life in married Turkish women. Sex Disabil.

[18] Stephenson KR, Meston CM (2010). Differentiating components of sexual well-being in women: are sexual satisfaction and sexual distress independent constructs?. J Sex Med.

[19] Magnay JL, Nevatte TM, O’Brien S, Gerlinger C, Seitz C (2014). Validation of a new menstrual pictogram (superabsorbent polymer-c version) for use with ultraslim towels that contain superabsorbent polymers. Fertil Steril.

[20] Symonds T, Boolell M, Quirk F (2005). Development of a questionnaire on sexual quality of life in women. J Sex Marital Ther.

[21] Mazokopakis E (2020). Sexual activity during menstruation in the holy Bible and Quran. Int J Fertil Steril.

[22] Mollazadeh S, Mirzaii Najmabadi K, Mirghafourvand M, Latifnejad Roudsari R (2023). Sexual activity during menstruation as a risk factor for endometriosis: a systematic review and meta-analysis. Int J Fertil Steril.

[23] McLaughlin SE, Ghanem KG, Zenilman JM, Griffiss JM (2019). Risk of gonococcal infection during vaginal exposure is associated with high vaginal ph and active menstruation. Sex Transm Dis.

[24] Silva AL Fo, Caetano C, Lahav A, Grandi G, Lamaita RM (2021). The difficult journey to treatment for women suffering from heavy menstrual bleeding: a multi-national survey. Eur J Contracept Reprod Health Care.

[25] Sinharoy SS, Chery L, Patrick M, Conrad A, Ramaswamy A, Stephen A (2023). Prevalence of heavy menstrual bleeding and associations with physical health and wellbeing in low-income and middle-income countries: a multinational cross-sectional study. Lancet Glob Health.

[26] Biscione A, Barra V, Bellone E, Severi FM, Luisi S (2020). Ulipristal acetate on quality of life and sexual function of women with uterine fibromatosis. Gynecol Endocrinol.

[27] Chang JH, Shin DW, Jeon MJ, Hong H, Kim SM, An A (2019). Uterine leiomyoma is associated with female sexual dysfunction in postmenopausal women. Yonsei Med J.

[28] Evruke I, Tas IS (2023). Operative Hysteroscopy Versus Levonorgestrel-Releasing Intrauterine System (LNG-IUS) insertion: a prospective observational study on the effect of surgical and medical treatments on sexual functions in women with abnormal uterine bleeding. Cureus.

[29] Vitale SG, Ferrero S, Ciebiera M, Barra F, Török P, Tesarik J (2020). Hysteroscopic endometrial resection vs. hysterectomy for abnormal uterine bleeding: impact on quality of life and sexuality. Evidence from a systematic review of randomized controlled trials. Curr Opin Obstet Gynecol.

[30] Abul R, Selcuki NFT, Karadeniz O, Bahat PY (2024). A comparison of the effects of oral dydrogesterone and levonorgestrel-releasing intrauterine device on quality of life and sexual function in patients with abnormal uterine bleeding. Arch Gynecol Obstet.

[31] Rosen R, Brown C, Heiman J, Leiblum S, Meston C, Shabsigh R (2000). The Female Sexual Function Index (FSFI): a multidimensional self-report instrument for the assessment of female sexual function. J Sex Marital Ther.

[32] Tuğut N, Gölbaşı Z, Ölçeği CYK (2010). Kadın Türkçe versiyonunun geçerlik ve güvenirlik çalışması. Cumhur Med J.

[33] Merghati-Khoei E, Emami-Razavi SH, Bakhtiyari M, Lamyian M, Hajmirzaei S, Ton-Tab Haghighi S (2017). Spinal cord injury and women’s sexual life: case-control study. Spinal Cord.

[34] Khastar H, Mirrezaie SM, Chashmi NA, Jahanfar S (2020). Sleep improvement effect on sexual life quality among rotating female shift workers: a randomized controlled trial. J Sex Med.

[35] Sharma JB, Kumari R, Kumari S, Jain S, Dharmendra S (2023). To study the efficacy and safety of diosmin with tranexamic acid and mefenamic acid versus only tranexamic acid and mefenamic acid in medical management of abnormal uterine bleeding: a randomized controlled trial. J Midlife Health.

[36] Soontrapa N, Rattanachaiyanont M, Warnnissorn M, Wongwananuruk T, Indhavivadhana S, Tanmahasamut P (2022). The effectiveness of desogestrel for endometrial protection in women with abnormal uterine bleeding-ovulatory dysfunction: a non-inferiority randomized controlled trial. Sci Rep.

[37] Hassanin AM, Kaddah AN, El-Amir MY (2019). The relationship of close marital affairs to healthy women’s sexual function: a cross-sectional retrospective study in Egypt. Sex Med.

[38] Dişli Çetinçay D, Bilgiç Fş (2026). Factors influencing premarital risky sexual behavior likelihood and human papilloma virus knowledge among young women: a cross-sectional study. Int J Gynaecol Obstet.

[39] Alimoradi Z, Ghorbani S, Bahrami N, Griffiths MD, Pakpour AH (2022). Socio-demographic predictors of dyadic sexual communication among Iranian married women. Sexologies.

[40] Rausch D, Rettenberger M (2021). Predictors of sexual satisfaction in women: a systematic review. Sex Med Rev.

[41] Momtaz YA, Hamid TA, Ibrahim R, Akahbar SAN (2014). Racial and socioeconomic disparities in sexual activity among older married Malaysians. Arch Gerontol Geriatr.

[42] Abdallah IY, ElHadi HM, Younis SI (2020). Are there differences in female sexuality related to educational level?. Benha J Appl Sci.

[43] Bagherinia M, Dolatian M, Mahmoodi Z, Ozgoli G, Alavi Majd H (2022). Evaluating the relationship between structural determinants of health and quality of sexual life in women: a systematic review. Crescent J Med Biol Sci.

[44] American College of Obstetricians and Gynecologists (2021). informed consent and shared decision making in obstetrics and gynecology: ACOG committee opinion, number 819. Obstet Gynecol.

[45] Young EJ, Kirk HM (2025). How women discuss heavy menstrual bleeding in online forums (“it’s like revenge of the uterus”): template approach to thematic analysis. J Med Internet Res.

[46] Pouraliroudbaneh S, Marino J, Riggs E, Saber A, Jayasinghe Y, Peate M (2024). Heavy menstrual bleeding and dysmenorrhea in adolescents: a systematic review of self-management strategies, quality of life, and unmet needs. Int J Gynaecol Obstet.

[47] Vannuccini S, Jain V, Critchley H, Petraglia F (2022). From menarche to menopause, heavy menstrual bleeding is the underrated compass in reproductive health. Fertil Steril.

[48] Kaphle M, Karki R, Regmi N, Poudyel P (2023). Social and cultural issues of menstruation and abnormal uterine bleeding in Nepal. Arch Obstet Gynaecol.

[49] Tan DA, Haththotuwa R, Fraser IS (2017). Cultural aspects and mythologies surrounding menstruation and abnormal uterine bleeding. Best Pract Res Clin Obstet Gynaecol.

[50] Vargün B (2021). Menstrüasyon ve tabu. Antropoloji.

[51] Hartman EA, Marston C, Namwebya J, Asala E, Ombidi W, Thungu F (2025). Intersections of menstruation, gender-based violence and contraceptive use: qualitative insights from girls and young women’s experiences in western Kenyan family planning clinics. BMJ Open.

[52] Eising H, Leijenaar E, Nasr R, Van Leuken R, Bongers M, Milota M (2025). Narrative medicine intervention on the obstetric–gynaecological work floor to discuss social stigmas around heavy menstrual bleeding using cocreated site-specific poetry. Med Humanit.

[53] Vallée A, Feki A, Ayoubi JM (2026). Using artificial intelligence to predict sexual health outcomes in endometriosis: a decision tree model algorithm. Sex Med.

[54] Clark TJ (2016). Heavy menstrual bleeding. InnovAiT.

[55] Sharma R (2017). Heavy menstrual bleeding: a hematology perspective - a short review. Open Access Blood Res Transfus J.

