# Supplementary material for: The effect of heavy menstrual bleeding on sexual quality of life: a case-control study
Source: Rev Esc Enferm USP. 2026 Sep 25;60:e20250041. doi: 10.1590/1980-220X-REEUSP-2025-0041en (PMC13619144; doi:10.1590/1980-220X-REEUSP-2025-0041en)
Supplement: Translation

# Translation: El efecto del sangrado menstrual abundante en la calidad de vida sexual: un estudio de casos y controles

**DOI:** 10.1590/1980-220X-REEUSP-2025-0041es

**Authors:** Aynur Kizilirmak, Pelin Calpbinici, Nur Șifa Yavuz

**Affiliations:** 1 Nevsehir Hacı Bektaş Veli University, Semra and Vefa Küçük Faculty of Health Sciences, Department of Obstetrics and Gynecology Nursing, Nevsehir, Turkey.

**Keywords:** Sexualidad, Menorragia, Mujeres

## Abstract

**Objetivo:**

Determinar el efecto del sangrado menstrual abundante (SMA) en la calidad de vida sexual de las mujeres.

**Método:**

Se llevó a cabo un estudio de casos y controles con un total de 142 mujeres (71 con SMA y 71 controles) que acudieron a las clínicas ambulatorias de un hospital público en Turquía. El grupo de casos estuvo compuesto por mujeres en edad reproductiva diagnosticadas con SMA, mientras que el grupo de control incluyó a mujeres sin SMA que cumplían con los criterios de inclusión. Los datos se recopilaron mediante un formulario de información personal y el cuestionario de Calidad de Vida Sexual para Mujeres (SQOL-F).

**Resultados:**

No se observaron diferencias significativas entre los grupos en cuanto a la edad y el índice de masa corporal (IMC) (p > 0,05). Sin embargo, en comparación con el grupo de control, las mujeres con SMA reportaron un mayor número de días de sangrado, coágulos menstruales, mayor uso de toallas sanitarias o tampones, mayor volumen de sangrado, molestias menstruales y ciclos menstruales más cortos (p < 0,05). Se observó una diferencia estadísticamente significativa entre los grupos en cuanto al nivel educativo (p < 0,005), mientras que no se encontró ninguna diferencia significativa en el uso de anticonceptivos (p > 0,05). La puntuación media del SQOL-F fue de 71,91 ± 20,60 para las mujeres con SMA y de 80,06 ± 17,39 para el grupo de control, lo que indica una diferencia estadísticamente significativa (p < 0,05). Además, el 26,8% de las mujeres con SMA presentaba una mala calidad de vida sexual.

**Conclusión:**

Aunque las diferencias en el nivel educativo pueden representar un posible factor de confusión, nuestros hallazgos demuestran que el sangrado menstrual abundante (SMA) reduce significativamente la calidad de vida sexual en las mujeres, lo que respalda nuestra hipótesis de investigación.

## INTRODUCCIÓN

La Federación Internacional de Ginecología y Obstetricia (FIGO) define el sangrado menstrual abundante (SMA) como la pérdida excesiva de sangre menstrual que afecta la calidad de vida en lo que respecta a la salud física, emocional, social y espiritual^(1,2)^. A pesar de la ausencia de un estudio poblacional exhaustivo para determinar la prevalencia del SMA, diversos estudios han documentado tasas de prevalencia que oscilan entre el 3% y el 40,14%^(3–8)^. Según la FIGO, el SMA se clasifica etiológicamente en causas estructurales PALM (pólipo, adenomiosis, leiomioma, neoplasia maligna) y causas no estructurales COEIN (coagulopatía, disfunción ovulatoria, endometrial, iatrogénica e inclasificable)^(9)^. Aunque se han reportado problemas de salud física como la pérdida excesiva de sangre debido al sangrado menstrual anormal, la anemia por deficiencia de hierro y la fatiga^(7)^, también se ha demostrado que afecta negativamente la calidad de vida, el nivel de energía y el estado de ánimo de las mujeres. Además, el sangrado menstrual anormal se ha relacionado con una reducción en la productividad laboral, las interacciones sociales, la vida familiar y la calidad de la vida sexual^(10,11)^.

La vida sexual de las mujeres ocupa un lugar significativo en sus vidas en general. Se estima que aproximadamente el 40% de las mujeres en edad reproductiva experimentan problemas de salud sexual^(12)^. Una gran variedad de factores puede influir en la función sexual de las mujeres, entre ellos la salud mental, las relaciones sexuales, la función sexual de la pareja, los factores relacionados con la personalidad, la duración de la relación, la infertilidad, los medicamentos, las enfermedades crónicas, la cirugía pélvica, los cánceres y los cambios posparto^(13,14)^. La sexualidad de una persona se ve significativamente influenciada por una gran variedad de factores, entre ellos su relación de pareja, el entorno cultural y social, las condiciones de vida, las características biológicas y la autopercepción. Además, las fluctuaciones hormonales, la menstruación, la lactancia, la menopausia y los partos múltiples pueden influir notablemente en el funcionamiento sexual de las mujeres^(15,16)^. Una revisión de la literatura revela que los términos “calidad de vida sexual”, “satisfacción sexual”, “felicidad sexual” y “bienestar sexual” se utilizan para expresar el mismo concepto subyacente^(17)^. En este estudio, se empleará el término “calidad de vida sexual” para evitar posibles contradicciones. El término “calidad de vida sexual” se define como “la evaluación subjetiva que hace una persona de los aspectos positivos y negativos de su relación sexual y su respuesta emocional posterior a dicha evaluación”^(18)^. La calidad de vida sexual está relacionada con los aspectos físicos, emocionales y relacionales de la sexualidad. A la luz de estas consideraciones, el placer sexual, el orgasmo y la frecuencia de las relaciones sexuales se consideran asociados con los aspectos físicos de la calidad de vida sexual^(17)^.

Se sabe que el sangrado menstrual abundante (SMA) es, en gran medida, sub-notificado. Los estudios indican que solo aproximadamente el 6% de las mujeres que padecen esta afección buscan atención médica^(4,19)^, lo cual puede atribuirse a percepciones erróneas sobre el SMA, la normalización de los síntomas y diversos factores culturales. En algunas culturas, el sangrado menstrual se percibe como una “limpieza médica” o se considera un signo de buena salud, mientras que las normas tradicionales de silencio pueden impedir que las mujeres obtengan información sobre la menstruación y busquen atención médica para los trastornos menstruales, al convertir dichos temas en tabú^(20)^.

En la mayoría de las principales religiones del mundo, está muy extendida la creencia de que se deben evitar las relaciones sexuales durante la menstruación^(21)^. La literatura también respalda esta perspectiva, al indicar que mantener relaciones sexuales durante este período puede provocar problemas de salud ginecológica, como la endometriosis^(22)^ e infecciones^(23)^. Por lo tanto, por lo general se evita la actividad sexual durante la menstruación. En los casos de SMA, el mayor número de días de sangrado y el flujo menstrual más abundante respaldan la hipótesis de que esto puede conducir a una reducción en la frecuencia de las relaciones sexuales. Además, la estructura multicultural de Turquía y la presencia de perspectivas similares dentro de la cultura turca enfatizan aún más la importancia de este estudio.

En este contexto, las enfermeras desempeñan un papel fundamental como primer punto de contacto con las pacientes en la identificación y el manejo del sangrado menstrual abundante, distinguiendo entre sangrado menstrual normal y anormal, brindando educación sobre el cuidado menstrual y reduciendo los tabúes relacionados con la menstruación^(24,25)^. Estas contribuciones de las enfermeras mejoran el conocimiento de las mujeres, facilitan el acceso a una atención adecuada y tienen el potencial de mejorar la calidad de vida sexual.

El sangrado menstrual abundante no solo impone una carga física; también puede afectar negativamente la calidad de vida relacionada con la salud, las interacciones sociales, la productividad laboral y la vida sexual^(10)^. La literatura existente incluye estudios que abordan el impacto del sangrado menstrual abundante y sus afecciones relacionadas en la sexualidad. En particular, se ha investigado el efecto del leiomioma uterino—una de las causas estructurales clasificadas según la clasificación PALM-COEIN—sobre la función sexual femenina^(26,27)^. Otros estudios han evaluado los cambios en la función sexual femenina tras tratamientos médicos y quirúrgicos administrados para el sangrado uterino anormal^(28–30)^. Sin embargo, en la gran mayoría de estos estudios, la función sexual femenina se evaluó mediante el Índice de Función Sexual Femenina (FSFI). Aunque la función sexual y la calidad de vida sexual están estrechamente relacionadas, constituyen conceptos distintos. Mientras que la función sexual se centra en los componentes fisiológicos de la respuesta sexual —como el deseo, la excitación, la lubricación, el orgasmo, la satisfacción y el dolor^(31)^—, la calidad de vida sexual refleja la satisfacción subjetiva y el bienestar psicosocial de una persona con respecto a su sexualidad^(20)^. Se llevó a cabo una búsqueda exhaustiva en las bases de datos PubMed, Scopus y Web of Science para identificar la evidencia existente en la literatura. Se utilizaron combinaciones de las palabras clave “sangrado menstrual abundante”, “sangrado uterino anormal”, “calidad de vida sexual”, “SQOL-F” y términos relacionados. Aunque varios estudios que investigan el sangrado menstrual abundante (SMA) han evaluado la función sexual femenina mediante el FSFI, no se pudo encontrar ningún estudio que evaluara el impacto del SMA en la calidad de vida sexual utilizando el cuestionario de Calidad de Vida Sexual para Mujeres (Sexual Quality of Life–Female-SQOL-F). Además, la promoción de la salud de las mujeres figura entre las prioridades fundamentales de los Objetivos de Desarrollo Sostenible (ODS) de las Naciones Unidas. En particular, el ODS 3 (Salud y bienestar) y el ODS 5 (Igualdad de género) hacen hincapié en el acceso universal a los servicios de salud sexual y reproductiva (metas 3.7 y 5.6). Determinar en qué medida el SMA afecta la calidad de vida sexual de las mujeres puede contribuir tanto al logro de estos objetivos globales como al desarrollo de intervenciones clínicas más efectivas. En consecuencia, este estudio tuvo como objetivo determinar el impacto del SMA en la calidad de vida sexual de las mujeres.

Se presentan las siguientes hipótesis para su consideración:

H0:
No existe diferencia en la calidad de vida sexual entre las mujeres con y sin SMA.
H1:
Existe una diferencia significativa en la calidad de vida sexual entre las mujeres con y sin SMA.

## MÉTODO

### Diseño del Estudio y Muestra

Se llevó a cabo un estudio de casos y controles para determinar el impacto del SMA en la calidad de vida sexual de las mujeres. Este estudio empleó un diseño prospectivo de casos y controles. El grupo de casos estuvo compuesto por mujeres en edad reproductiva que acudieron a la clínica ambulatoria de obstetricia y ginecología de un hospital estatal en una provincia de Turquía y a quienes un profesional de la salud calificado les diagnosticó SMA. Para garantizar la privacidad y la confidencialidad, los datos se recopilaron en un entorno tranquilo y privado. Las mujeres del grupo con SMA completaron los cuestionarios en una sala privada de procedimientos ginecológicos, mientras que las del grupo de control lo hicieron en una sala privada designada para tal fin. Solo la participante y el investigador estuvieron presentes durante la recopilación de datos, y el investigador se encontraba disponible únicamente para aclarar cualquier duda relacionada con los cuestionarios. Todos los cuestionarios se completaron antes de la consulta de las participantes con el médico. Se recopilaron datos descriptivos de las participantes mediante un formulario de información basado en los criterios del ACOG (American College of Obstetricians and Gynecologists) para el SMA. La coherencia de los hallazgos con la definición del ACOG indica que las participantes del grupo de casos fueron identificadas con precisión. El grupo de control estuvo compuesto por mujeres en edad reproductiva que acudieron a otras clínicas ambulatorias del mismo hospital por diversas razones, cumplían con los criterios de inclusión y no tenían diagnóstico de SMA.

*Criterios de inclusión:* se incluyeron en el estudio mujeres sexualmente activas de 18 años o más. *Criterios de exclusión:* se excluyeron del estudio las mujeres menopáusicas, aquellas con trastornos mentales, con discapacidades que causaran dificultades de comunicación (como deficiencias auditivas, del habla o del lenguaje) y sin enfermedades crónicas. El tamaño de la muestra se calculó utilizando el software de análisis estadístico OpenEpi Versión 3.01 (http://www.openepi.com). Se calculó un tamaño de muestra de al menos 64 mujeres en cada grupo utilizando un margen de error del 5%, una potencia del 90%, una proporción de casos y controles de 1:1, una razón de probabilidades del 3,5% y una prevalencia de SMA del 27,2%^(4)^. Se alcanzó el tamaño de muestra requerido mediante la inclusión de 71 mujeres de los grupos de casos y controles, lo que permitió la posible inclusión de participantes ausentes.

### Herramientas de Recopilación de Datos

Los investigadores utilizaron el Formulario de Información Descriptiva y la SQOL-F para la recopilación de datos.

*Formulario de Información Descriptiva:* Los investigadores desarrollaron el formulario de acuerdo con la literatura existente sobre el tema. Consistía en 20 preguntas que evaluaban tanto las características sociodemográficas (edad, nivel educativo, ocupación, nivel de ingresos, etc.) como las características ginecológicas (edad de la primera menarquia, características menstruales, uso de anticonceptivos, etc.).

*Escala de Calidad de Vida Sexual para Mujeres (SQOL-F):* La escala desarrollada por Symonds et al.^(20)^ fue adaptada al turco por Tuğut y Gölbaşı^(32)^. Esta escala tipo Likert de 6 puntos cuenta con 18 ítems. Se espera que las encuestadas respondan cada ítem basándose en sus experiencias sexuales de las últimas cuatro semanas. En el sistema de puntuación, a cada ítem se le asigna un valor entre 1 y 6, lo que permite una puntuación total entre 18 y 108; una puntuación alta indica una calidad positiva de la vida sexual. Esta medida es un instrumento útil para evaluar la calidad de la vida sexual de las mujeres, con clasificaciones categóricas de deficiente (rango de puntuación: 18–51), moderada (rango de puntuación: 51–84) y buena (rango de puntuación: 84)^(33)^. En la adaptación turca de la escala, el valor alfa de Cronbach fue de 0,83^(32)^. En el presente estudio, se determinó que el valor del alfa de Cronbach era de 0,89.

### Recolección de Datos

Los datos se recolectaron entre junio de 2023 y enero de 2024 mediante entrevistas presenciales con las participantes de los grupos de caso y de control. Se obtuvo el consentimiento informado antes de la recolección de datos. La duración de la entrevista osciló entre 10 y 15 minutos.

### Aprobación Ética

Se obtuvo la aprobación ética del Comité de Ética en Investigación Clínica No Intervencionista de la universidad correspondiente antes de iniciar la investigación (Fecha: 27/03/2023, N.º de decisión: 2023.05.09). Se obtuvo autorización por escrito (Fecha: 24/05/2023, N.º de decisión: E-26171210-929-216207373) de la institución pertinente para llevar a cabo la investigación. Además, se informó a las mujeres que participaron en el estudio sobre el propósito, los posibles beneficios y el tiempo estimado necesario para la entrevista. Esta información se les proporcionó antes de la entrevista y se obtuvo su consentimiento por escrito.

### Análisis Estadístico

Los datos se analizaron utilizando el paquete de software IBM SPSS 25.0 (Statistical Package for the Social Sciences). Los análisis estadísticos incluyeron el cálculo de distribuciones porcentuales, la determinación de medias aritméticas, el cálculo de desviaciones estándar, la realización de pruebas t en grupos independientes, la aplicación de pruebas de chi-cuadrado y la realización de análisis de confiabilidad con el alfa de Cronbach. Los resultados se analizaron con un intervalo de confianza del 95%, y se aceptó la significación estadística a un nivel de p < 0,05.

## RESULTADOS

Se incluyeron en este estudio un total de 142 mujeres, que comprendían 71 casos de SMA y 71 controles. No se observó ninguna diferencia estadísticamente significativa entre la edad promedio y el índice de masa corporal (IMC) de los dos grupos (p > 0,05) (Tabla 1). El nivel educativo de las mujeres del grupo de control fue significativamente más alto, y se identificó una diferencia estadísticamente significativa en el nivel educativo entre los grupos (p < 0,05; Tabla 1).

**Tabla 1:** Comparación de las características sociodemográficas entre los grupos de casos y de control (Nevşehir, Turquía, 2024).

Variable	Caso (n = 71)	Grupo de control (n = 71)	Análisis estadístico	Valor *p*
	n (%)	n (%)		
**Edad (media ± DE)**	44,41 ± 5,19	42,49 ± 7,26	1,809*	0,073
**Nivel educativo**				
Alfabetizados/escolaridad primaria	38 (53,5)	21 (29,6)	15,024**	**0,001**
Educación secundaria/bachillerato	22 (31,0)	19 (26,8)		
Universidad	11 (15,5)	31 (43,7)		
**Situación económica**				
Ingresos inferiores a los gastos	5 (7,0)	16 (22,5)	9,008**	**0,011**
Ingresos iguales a los gastos	61 (85,9)	46 (64,8)		
Ingresos superiores a los gastos	5 (7,0)	9 (12,7)		
**IMC (media ± DE)**	75,28 ± 13,12	72,63 ± 13,45	1,188*	0,23

*t: prueba t independiente

**X^2^: prueba de chi-cuadrado

Las mujeres con SMA presentaron una duración del sangrado significativamente mayor, coágulos menstruales más frecuentes, intervalos entre ciclos menstruales más cortos, un sangrado menstrual más abundante, sangrados excesivos molestos durante la menstruación más frecuentes, más pérdidas de sangre en la cama o en la ropa interior y más sangrados intermenstruales que las mujeres del grupo de control (todos p < 0,05; Tabla 2).

**Tabla 2:** Comparación de los signos y síntomas menstruales entre mujeres con y sin SMA (Nevşehir, Turquía, 2024).

Variable	Caso (n = 71)	Grupo de control (n = 71)	Análisis estadístico	Valor *p*
	n (%)	n (%)		
**Edad de la menarquia (media ± DE)**	13,29 ± 1,35	13,35 ± 1,08	–0,275	0,784
**Número de días de sangrado**				
1–7	41 (57,7)	62 (87,3)	15,589	**<0,001**
8 o más	30 (42,3)	9 (12,7)		
**Coágulos de sangre menstrual**				
Sí	55 (77,5)	27 (38,0)	22,628	**<0,001**
No	16 (22,5)	44 (62,0)		
**Duración del ciclo menstrual (días)**				
8–24	46 (64,8)	16 (22,5)	25,766	**<0,001**
24 y más	25 (35,2)	55 (77,5)		
**Cantidad de sangrado menstrual**				
Abundante	48 (67,6)	7 (9,9)	28,873	
Moderado	23 (32,4)	61 (85,9)		**<0,001**
Leve	–	3 (4,2)		
**Sangrado excesivo molesto durante la menstruación**				
Sí	54 (76,1)	19 (26,8)	34,534	**<0,001**
No	17 (23,9)	52 (73,2)		
**Pérdida de sangre en la ropa de cama o la ropa interior**				
Sí	55 (77,5)	21 (29,6)	32,726	**<0,001**
No	16 (22,5)	50 (70,4)		
**Sangrado intermenstrual**				
Sí	35 (49,3)	10 (14,1)	20,332	**<0,001**
No	36 (50,7)	61 (85,9)		

Las mujeres con SMA también indicaron que utilizaban un mayor número de compresas o tampones al día, que recurrían con mayor frecuencia al uso de doble protección higiénica y que se despertaban más a menudo por la noche para cambiarse la protección higiénica que las mujeres del grupo de control (todas p < 0,05; Tabla 3). No se observaron diferencias estadísticamente significativas entre los grupos en cuanto al uso de anticonceptivos o a los métodos anticonceptivos (p > 0,05; Tabla 3).

**Tabla 3:** Comparación de los hábitos de higiene menstrual y las características anticonceptivas entre mujeres con y sin SMA (Nevşehir, Turquía, 2024).

Variable	Caso (n = 71)	Grupo de control (n = 71)	Análisis estadístico	Valor *p*
	n (%)	n (%)		
**Número de compresas o tampones al día**				
1–4	41 (57,7)	56 (78,9)	7,320	**0,006**
5–10	30 (42,3)	15 (21,1)		
**Uso de compresas dobles durante la menstruación**				
Sí	35 (49,3)	10 (14,1)	20,332	**<0,001**
No	36 (50,7)	61 (85,9)		
**Despertarse por la noche para cambiarse las compresas durante la menstruación**				
Sí	48 (67,6)	20 (28,2)	22,124	**<0,001**
No	23 (32,4)	51 (71,8)		
**Uso de anticonceptivos**				
Sí	48 (67,6)	54 (76,1)	1,253	0,263
No	23 (32,4)	17 (23,9)		
**Métodos anticonceptivos**				
Anticonceptivos orales combinados / anticonceptivos inyectables mensuales / dispositivo intrauterino hormonal (DIU-LNG)	3 (6,3)	6 (11,1)	9,375	0,051
DIU de cobre (no hormonal)	13 (27,1)	11 (20,4)		
Condón masculino	18 (37,5)	12 (22,2)		
Ligadura de trompas	10 (20,8)	9 (16,7)		
Coito interrumpido	4 (8,3)	16 (29,6)		

La puntuación media en la escala SQOL-F de las mujeres con SMA fue de 71,91 ± 20,60, mientras que la puntuación media de las mujeres sin SMA fue de 80,06 ± 17,39. Se observó una diferencia significativa entre las puntuaciones medias en la escala SQOL-F de los grupos de casos y de control (p < 0,05). Un total de 19 (26,8%) mujeres con SMA presentaron una SQOL-F deficiente, 27 (38,0%) mostraron una SQOL-F moderada y 25 (35,2%) presentaron una SQOL-F buena. La comparación entre los grupos de casos y de control reveló que las mujeres con SMA presentaban una menor calidad de vida sexual (p < 0,05; Tabla 4).

**Tabla 4:** Comparación de las puntuaciones medias de la Escala de Calidad de Vida Sexual en los grupos de casos y de control (Nevşehir, Turquía, 2024).

SQOL-F	Caso (n = 71)	Grupo de control (n = 71)	Análisis estadístico	Valor *p*
SQOL-F (media ± DE)	71,91 ± 20,60	80,06 ± 17,39	–2,548	**0,012**
Deficiente	19 (26,8)	8 (11,3)	6,465	**0,039**
Moderado	27 (38,0)	27 (38,0)		
Bueno	25 (35,2)	36 (50,7)		

## DISCUSIÓN

Aunque en gran medida no se reporta lo suficiente^(4)^, en la literatura se documenta que más de un tercio de las mujeres en edad reproductiva experimentan sangrado menstrual irregular^(7)^. Dada la posible influencia de la edad tanto en el sangrado menstrual irregular como en la calidad de vida sexual, las características de edad de los grupos de casos y de control en nuestro estudio fueron similares. En la literatura existente, los estudios sobre temas similares comparan sistemáticamente a los grupos según sus características de edad^(34–36)^.

La mayor proporción de mujeres con título universitario en el grupo de control indica una diferencia estadísticamente significativa en el nivel educativo entre los grupos, lo cual podría actuar como un posible factor de confusión que afecte la calidad de vida sexual de las mujeres. La educación es un determinante clave en la configuración de la salud sexual, el deseo sexual y los niveles de actividad sexual de las personas^(37)^. Los niveles más altos de educación se asocian con el desarrollo de hábitos de vida más saludables, un mayor acceso a los servicios de salud y una mayor probabilidad de obtener información precisa sobre la salud sexual^(38)^. La educación superior está relacionada con mejores habilidades de comunicación, lo que puede fortalecer la comunicación sexual y la satisfacción dentro de las relaciones^(39)^. Sin embargo, un mayor nivel educativo también puede exponer a las personas a condiciones laborales más estresantes y exigentes, lo que puede afectar negativamente la satisfacción sexual debido al aumento del estrés y a los pensamientos que distraen^(40)^. Además, las personas con distintos niveles educativos pueden presentar prioridades y expectativas significativamente diferentes con respecto a la actividad sexual^(41)^. Un estudio realizado con 300 mujeres casadas que evaluó el impacto de la educación en la sexualidad femenina^(42)^ encontró una relación significativa entre el nivel educativo y la calidad de vida sexual. Este hallazgo concuerda con una revisión sistemática que examinó los determinantes estructurales de la salud, la cual, de manera similar, informó una asociación entre la educación y la calidad de vida sexual de las mujeres^(43)^. Aunque es difícil “establecer” límites claros entre la educación y la sexualidad, las personas con niveles educativos más altos generalmente reportan mejores resultados de salud sexual, poseen una perspectiva más amplia sobre el bienestar sexual y experimentan una mayor satisfacción general con sus vidas sexuales^(39)^. Cuando se analizan de manera conjunta los hallazgos derivados de la literatura, se hace evidente que el efecto del nivel educativo sobre la calidad de vida sexual es multidimensional y puede manifestarse de diversas formas, dependiendo de las condiciones de vida de la persona, la dinámica de sus relaciones y el contexto cultural. En consecuencia, el desequilibrio en la distribución educativa entre los grupos del presente estudio se presenta como una consideración metodológica importante que justifica una interpretación cautelosa de los resultados obtenidos. Se recomienda que futuras investigaciones aborden este tema mediante procedimientos de emparejamiento o controles estadísticos.

En 2021, el Colegio Americano de Obstetras y Ginecólogos (American College of Obstetricians and Gynecologists – ACOG) definió el SMA como un sangrado que persiste durante más de siete días, empapa uno o más tampones o toallas sanitarias cada hora durante varias horas consecutivas, requiere el uso de más de una toalla sanitaria a la vez para controlar el flujo, exige cambiar las toallas sanitarias o los tampones durante la noche e incluye coágulos de sangre de un cuarto de tamaño o más^(44)^. El grupo de casos estuvo compuesto por mujeres a quienes un profesional médico calificado les había diagnosticado SMA. En nuestro estudio, las mujeres con SMA presentaron una mayor prevalencia de los siguientes síntomas en comparación con el grupo de control: mayor duración del sangrado, coágulos menstruales, uso de toallas sanitarias o tampones por día, volumen del sangrado menstrual, malestar debido al sangrado excesivo durante la menstruación, uso de dos toallas sanitarias a la vez durante la menstruación, manchado en la cama o en la ropa interior durante la menstruación, cambios nocturnos de toallas sanitarias durante la menstruación y una mayor frecuencia de la menstruación. Nuestros hallazgos concuerdan con la definición de sangrado menstrual abundante (SMA) del ACOG^(44)^. Esto sugiere que las participantes del grupo de casos fueron identificadas correctamente.

Nuestro estudio reveló que las mujeres con SMA reportaron una mayor prevalencia de duración prolongada del sangrado, mayor volumen de sangrado, coágulos de sangre, uso de toallas sanitarias dobles, cambios nocturnos de toallas sanitarias y síntomas que limitan la vida social en comparación con el grupo de control.

Este hallazgo es significativo, ya que la literatura indica que el sangrado menstrual abundante afecta negativamente el bienestar social, emocional y psicológico de las mujeres, lo que perjudica directa e indirectamente la calidad de vida sexual. El sangrado menstrual abundante restringe la participación en actividades sociales y de ocio y limita la actividad física debido al temor a las fugas y a la necesidad frecuente de cambiar los productos sanitarios^(45)^. Estos factores contribuyen a la angustia emocional, incluyendo el bajo estado de ánimo, la ansiedad y la reducción de la calidad de vida. Dado que la salud emocional está estrechamente vinculada a la calidad de vida sexual, la carga psicológica asociada con el sangrado menstrual abundante puede afectar negativamente el deseo y la satisfacción sexuales^(46,47)^. Además, la naturaleza crónica del sangrado menstrual abundante puede conducir a estrés psicológico a largo plazo, lo que a su vez puede afectar negativamente las relaciones íntimas^(45)^. Por consiguiente, el SMAno debe considerarse simplemente como un trastorno fisiológico de sangrado, sino como una entidad clínica integral que ejerce efectos adversos medibles sobre la salud sexual, el funcionamiento emocional y la calidad de las relaciones. En este sentido, el manejo clínico de el SMArequiere el desarrollo y la implementación de modelos de intervención multidisciplinarios que, junto con el tratamiento sintomático, se enfoquen explícitamente en el bienestar sexual y la salud psicosocial de las mujeres. Aunque las mujeres con SMA informaron un mayor uso de toallas sanitarias o tampones y cambios nocturnos más frecuentes de las toallas, estos comportamientos también pueden verse influenciados por las prácticas de higiene individuales, las preferencias personales o la capacidad de absorción de los productos sanitarios. Por lo tanto, estas variables no deben interpretarse como indicadores independientes de la pérdida de sangre menstrual, sino más bien como hallazgos conductuales de apoyo que deben considerarse junto con el diagnóstico de SMA confirmado por un médico y otras características clínicas.

La experiencia subjetiva de el SMAestá determinada por las normas culturales y las expectativas sociales. En muchas culturas donde la menstruación es estigmatizada, las mujeres pueden sentirse presionadas a ocultar sus síntomas, lo que puede influir negativamente en su participación en la actividad sexual y en su disposición a hablar sobre sus inquietudes de salud sexual con sus parejas o con profesionales de la salud^(48)^. Los tabúes menstruales, los sentimientos de vergüenza y la percepción de estar “sucias” pueden llevar a las mujeres a evitar la intimidad sexual y a dudar en buscar atención médica, lo que afecta negativamente tanto a la calidad de vida sexual como al bienestar general^(48–50)^. Esta situación provoca retrasos en el diagnóstico y una carga prolongada de los síntomas, lo que contribuye a la cronificación del malestar psicológico y, en consecuencia, a un mayor deterioro de la calidad de vida sexual.

El sangrado menstrual abundante está estrechamente asociado con el afecto negativo, la vergüenza social y la disminución de la moral. El riesgo de sangrado abundante y el miedo a las fugas llevan a las mujeres a aislarse no solo de los entornos sociales, sino también de las relaciones íntimas, lo que puede resultar en un debilitamiento de la autoconfianza sexual y una reducción del deseo de participar en actividades sexuales^(49)^. De hecho, la literatura actual demuestra que el sangrado menstrual abundante (SMA) se vive como una limitación sexual y ejerce efectos adversos directos sobre la autonomía sexual de las mujeres^(51)^. Además, el estigma social específico del sangrado menstrual abundante dificulta que las mujeres expresen abiertamente esta experiencia, lo que constituye una barrera para la comunicación sexual tanto con los profesionales de la salud como con sus parejas^(52)^. En consecuencia, el sangrado menstrual abundante puede afectar sustancialmente la calidad de vida sexual no solo a través de mecanismos fisiológicos, sino también a través de profundas vías sociales y emocionales.

Se observó que los puntajes promedio de la escala SQOL, tanto para el grupo de casos como para el de control, se encontraban en un nivel intermedio. Sin embargo, se observó que el puntaje promedio de la escala SQOL era más bajo en las mujeres con SMA en comparación con las mujeres sin SMA. Una revisión de la literatura revela una escasez de estudios directos que evalúen la calidad de vida sexual entre las mujeres con SMA. No obstante, un metaanálisis de estudios con mujeres en edad reproductiva reveló que la presencia de una enfermedad crónica era un predictor significativo de la calidad de vida sexual^(13)^. Vallée et al.^(53)^ identificaron el sangrado menstrual anormal como un predictor independiente y sólido de la disfunción sexual en las mujeres, y señalaron que su presencia, junto con los cólicos menstruales intensos y la ausencia de tratamiento, aumenta significativamente el riesgo de disfunción sexual.

Por el contrario, la revisión sistemática realizada por Vitale et al.^(29)^ demostró que los tratamientos dirigidos al sangrado uterino anormal produjeron mejoras significativas en la calidad de vida sexual y el bienestar psicológico, lo que corrobora indirectamente el impacto adverso del sangrado uterino anormal no tratado sobre la calidad de vida sexual. El estudio reveló que una de cada cuatro mujeres con sangrado uterino anormal y una de cada diez mujeres sin sangrado uterino anormal presentaban un nivel deficiente de calidad de vida sexual. Por el contrario, aproximadamente una de cada tres mujeres con sangrado uterino anormal y una de cada dos mujeres sin este sangrado mostraron un nivel favorable de calidad de vida sexual (Tabla 3). Los resultados de nuestra investigación indican que las mujeres que padecen sangrado uterino anormal reportan una menor calidad de vida sexual que las mujeres que no padecen dicho sangrado. Los resultados de nuestro estudio concuerdan con nuestra hipótesis de investigación inicial.

Las enfermeras desempeñan un papel fundamental en la identificación, el manejo y la educación relacionados con el SMA^(54)^. Evaluar tanto los efectos directos como los indirectos de el SMAen la calidad de vida sexual es un componente esencial de la atención de enfermería. Al brindar a las mujeres información sobre las causas, los síntomas y las estrategias de autocontrol de la SMA, las enfermeras pueden ayudarlas a distinguir entre ciclos menstruales normales y anormales^(55)^. Fomentar la comunicación abierta sobre la salud sexual, ofrecer derivaciones para asesoramiento psicológico y brindar orientación para reducir los tabúes sociales se encuentran entre las contribuciones significativas de las enfermeras para mejorar la calidad de vida sexual de las mujeres^(24,54,55)^. Por lo tanto, las enfermeras ocupan una posición clave para mitigar el impacto de el SMAen la salud sexual.

### Fortalezas y Limitaciones del Estudio

La fortaleza de este estudio radica en que el grupo de casos fue diagnosticado por un médico, con el respaldo de los criterios de diagnóstico del SMA establecidos por el ACOG. Sin embargo, entre las limitaciones se incluyen el emparejamiento de los grupos únicamente por edad y el hecho de basarse en respuestas a cuestionarios autoinformadas. Además, la diferencia estadísticamente significativa en el nivel educativo entre los grupos puede representar un factor de confusión que influya en las percepciones de la calidad de vida sexual.

## CONCLUSIÓN

A pesar de la diferencia estadísticamente significativa en el nivel educativo entre los grupos de casos y de control, y de considerar esta diferencia como un posible factor de confusión que afecta la calidad de vida sexual, nuestros hallazgos demuestran que las mujeres con SMA tienen una calidad de vida sexual significativamente menor en comparación con aquellas sin SMA, lo que respalda nuestra hipótesis de investigación.

## Data Availability

El conjunto de datos que respaldan las conclusiones de este estudio está disponible previa solicitud al autor correspondiente.
